# CRISPR/Cas: An Emerging Toolbox for Engineering Virus Resistance in Plants

**DOI:** 10.3390/plants13233313

**Published:** 2024-11-26

**Authors:** Xiaohui Zhan, Fengjuan Zhang, Ning Li, Kai Xu, Xiaodi Wang, Shenghua Gao, Yanxu Yin, Weiling Yuan, Weifang Chen, Zhiyong Ren, Minghua Yao, Fei Wang

**Affiliations:** 1 Hubei Key Laboratory of Vegetable Germplasm Innovation and Genetic Improvement, Cash Crops Research Institute, Hubei Academy of Agricultural Sciences, Wuhan 430062, China; 13971406680@163.com (X.Z.); li_ning@hbaas.com (N.L.); kaixu@hbaas.com (K.X.); 15927361502@163.com (X.W.); gaoshenghua1986@126.com (S.G.); yinyanxu2008@126.com (Y.Y.); ywiing2021@hbaas.com (W.Y.); wfchen0719@hbaas.com (W.C.); rzy@hbaas.com (Z.R.); 2 State Key Laboratory of Biocatalysis and Enzyme Engineering, School of Life Sciences, Hubei University, Wuhan 430062, China; zhangfengjuan159@163.com; 3 Hubei Hongshan Laboratory, Wuhan 430070, China

**Keywords:** CRISPR/Cas9, CRISPR/Cas13, virus resistance, *Geminivirus*, *Potyvirus*, *eIF4E*

## Abstract

Clustered regularly interspaced short palindromic repeats (CRISPR)/Cas have been recognized as powerful genome-editing tools in diverse eukaryotic species, including plants, and thus hold great promise for engineering virus resistance in plants. Nevertheless, further attention is required regarding various issues associated with applying new powerful technologies in the field. This mini-review focuses on the recent advances in using CRISPR/Cas9 and CRISPR/Cas13 systems to combat DNA and RNA viruses in plants. We explored the utility of CRISPR/Cas for targeting the viral genome and editing host susceptibility genes in plants. We also provide insights into the limitations and challenges of using CRISPR/Cas for plant virus interference and propose individual combinatorial solutions. In conclusion, CRISPR/Cas technology has the potential to offer innovative and highly efficient approaches for controlling viruses in important crops in the near future.

## 1. Introduction

Natural CRISPR/Cas (clustered regularly interspaced short palindromic repeats and CRISPR-associated) systems are adaptive immune mechanisms in prokaryotes that provide protection against phages and other invasive genetic elements [1]. This system cleaves foreign nucleic acids as RNA-guided endonucleases based on sequence complementarity. CRISPR/Cas systems are classified into two different classes, six types, and several subtypes on the basis of the genomic architecture of the CRISPR array and signature Cas genes [2,3]. Among these, Class I (Types I, III, and IV) uses multi-Cas subunits for nucleic acid interference, whereas Class II (Types II, V, and VI) requires only one efficient single CRISPR-Cas effector complexed with guide RNAs (gRNAs) [4,5]. Based on their high target precision, simplicity, efficiency, and multiplexing capabilities, Class II CRISPR/Cas systems have been widely adopted as promising gene editing tools to control plant viral diseases and accelerate plant protection.

Plant viruses can infect a diverse range of economically important crops, contributing to significant losses of approximately 10–15% of global plants each year [6,7]. Plant viruses are classified into six major groups based on their genomic structures: double-stranded DNA (dsDNA) viruses, single-stranded DNA (ssDNA) viruses, reverse-transcribing viruses, double-stranded RNA (dsRNA) viruses, positive-sense single-stranded RNA (+ssRNA) viruses, and negative-sense single-stranded RNA (−ssRNA) viruses [8,9]. Most investigations using the CRISPR/Cas system to combat plant viruses have primarily focused on ssDNA *Geminiviruses* and ssRNA *Potyviruses*. Consequently, this review predominantly discusses the progress made in studies targeting *Geminiviruses* and *Potyviruses*.

*Geminiviruses*, a type of ssDNA virus, cause significant losses in various important plants. The genus *Begomovirus* is the most important member of *Geminiviruses* [8]. *Begomoviruses* infect various important dicotyledonous plants and are primarily transmitted through insect vectors, particularly whiteflies [10]. *Begomovirus* is divided into monopartite (DNA-A) and bipartite (DNA-A and DNA-B) components.

The *Potyvirus* is the largest genus in the family *Potyviridae*, the largest group of RNA plant viruses. This genus is known to infect a wide range of plant species [11,12]. It causes substantial harvest losses worldwide [12] through mechanical transmission or vector (aphid) transmission in a non-circulative, non-persistent, and stylet-borne manner.

Enhancing plant resistance to control plant viruses includes conventional resistance breeding, RNA silencing, and transgenic plants over the past years [12]. Conventional resistance breeding strategies using dominant resistance genes are a promising option and have been most commonly used in practical production. To date, numerous dominant resistance genes have been reported in major vegetable crops [13]. RNA silencing, referred to as RNA interference (RNAi), is a highly efficient and successful approach for generating transgenic antiviral RNAi plants. To date, RNAi technology has successfully controlled more than 60 economically important plant viruses [14]. Transgenic plants also achieve high levels of resistance, especially when expressing the viral coat protein (CP) of viruses [15]. Despite these efforts, the development of resistance to these viruses has been limited. Therefore, robust and highly efficient resistance strategies are required. Recently, CRISPR/Cas systems have been used to engineer virus resistance in a broad spectrum of plants. These novel technologies have created new dimensions for developing potential antiviral tools to combat devastating viruses. In this mini-review, we discuss recent advances in virus resistance achieved using the CRISPR/Cas system in diverse plant species. Furthermore, we discuss the challenges encountered in deploying CRISPR/Cas-mediated defenses against plant viruses. Finally, we present perspectives for future research.

## 2. CRISPR/Cas for Plant Genome Editing

In many plant species, CRISPR/Cas systems offer a versatile toolkit for sequence-specific manipulation, including targeted mutagenesis, multiplex editing, trait modifications, gene replacement, gene knock-in, and pathogen detection [5,16]. As site-directed genome-editing tools, CRISPR–Cas9, Cas12a, and Cas12b have become widely used sequence-specific nucleases in plant genome engineering [16,17]. Similarly, CRISPR-Cas13 can be used for targeted RNA cleavage and downregulation of specific transcripts [18,19,20]. Advances in the Class II system for genome editing have been recognized as an efficient gene modification technology for making virus-resistant plants.

## 3. Engineering Virus Resistance in Plants Based on CRISPR/Cas

**Figure 1:**
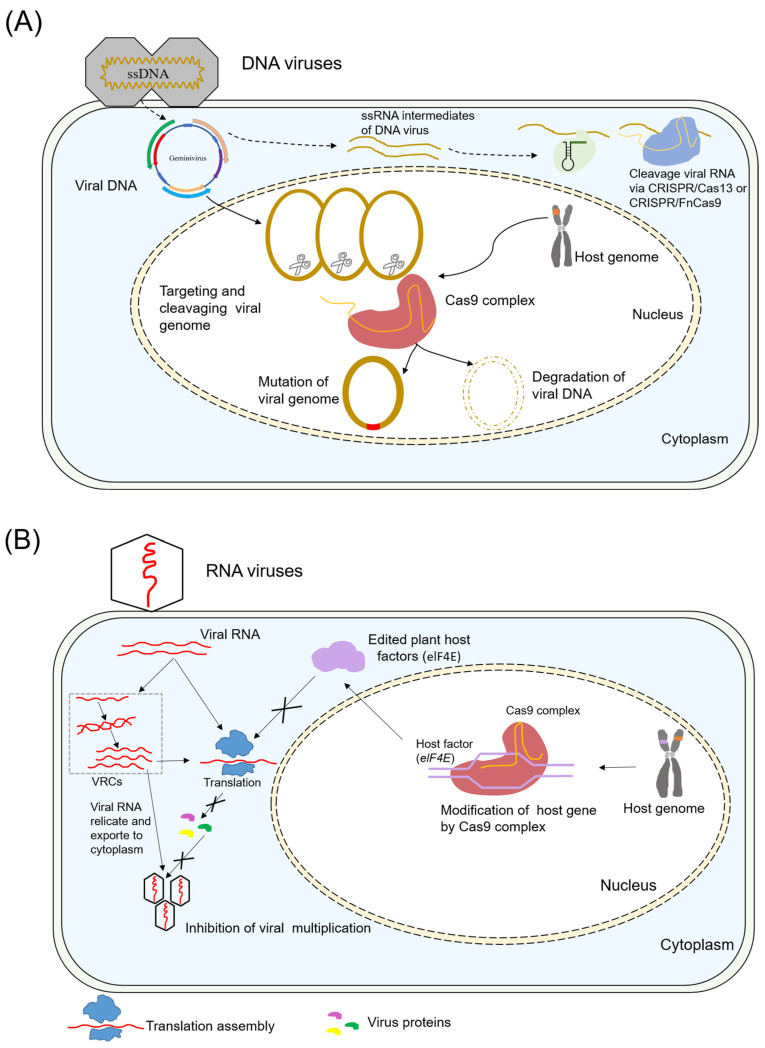

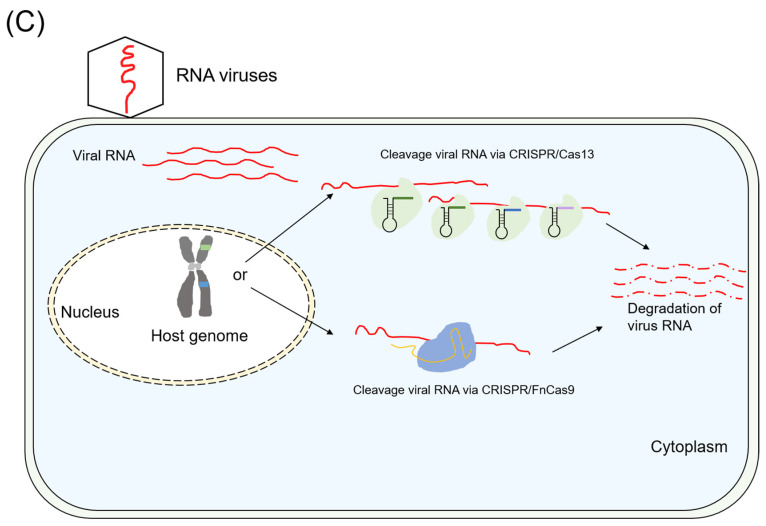
Schematic diagram of Class 2 CRISPR/Cas strategies for combating viruses in plants is presented. (**A**) Representation of the CRISPR/Cas9 mechanism to confer resistance against DNA viruses. The Cas9/sgRNA complex originating from the host genome binds to and cleaves the viral genome regions when DNA viruses invade plant cells. Direct targeting of the viral genome sequence can modulate the mutation and degradation processes. Alternatively, both FnCas9 and Cas13a effectors have been designed to target and cleave viral RNA transcripts, which are intermediates produced by DNA viruses. (**B**) Overview of RNA virus resistance mediated by CRISPR/Cas9 targeting host genes. RNA viruses interact with host susceptibility factors to facilitate their infection. VRCs: the viral replication complex, which provides an optimal, protective microenvironment for efficient viral RNA replication (**C**). Similarly, for RNA viruses, FnCas9 and Cas13a can be engineered to target and cleave the viral genome or transcripts, leading to degradation of the RNA viral genome.

Protection against viruses is divided into two different strategies: (a) directly targeting the viral genome for cleavage or degradation and (b) targeting host factors essential in the virus life cycle for mutation (Figure 1).

### 3.1. Virus Interference by Targeting of the Virus Genome in Plants via CRISPR/Cas9

The CRISPR/Cas system, a defense mechanism in bacteria and archaea against plasmids and invading phages [3,21], has proven to be a highly efficient genome-editing tool in diverse eukaryotic species. The significant advantage of CRISPR/Cas technology is that sgRNA sequences can easily be reprogrammed to edit targets, and numerous gRNAs can be expressed simultaneously with a single Cas9 protein to target multiple sites of the target genome [22,23]. Consequently, CRISPR/Cas9-based gene editing has been effectively used to tolerate diverse viral DNA infections in plants (Figure 1A).

CRISPR/Cas9 systems have been used to minimize viral DNA by disrupting the viral genomes of plant cells [24,25,26] (Table 1). Ji et al. (2015) demonstrated how the CRISPR/Cas9 system could confer resistance to beet-severe curly top virus (BSCTV) in transgenic plants by targeting Rep, CP, and IR for mutagenesis [27]. Concurrently, this system exhibits prominent resistance to bean yellow dwarf virus (BeYDV) in *N. benthamiana* [28].

In line with these findings, Ali et al. (2015) also demonstrated that the CRISPR/Cas9 system could be engineered to interfere with multiple DNA viruses in transgenic plants by designing gRNAs against conserved sequences in the viral intergenic region that is conserved, pointing to the feasibility of combating multiple viruses with one system [29]. Continued research has revealed that viruses can escape the antiviral CRISPR transgene through single-nucleotide mutations in the genome. Ali et al. (2016) further indicated that it is feasible to engineer this system to target the non-coding regions of the *Geminiviruses*, limiting viral escape more effectively than by targeting the coding regions [30], and inferred that the non-coding regions of the viral genome could potentially serve as superior targets for engineering plant resistance to DNA viruses.

Several studies have shown that Cas9 equipped with dual gRNAs can be engineered to cleave the DNA genome. The amalgamation of two or more gRNAs with the CRISPR/Cas9 system could enhance its mediated immunity against cotton leaf curl Multan virus (CLCuMuV) [31]. Roy et al. (2019) also pointed to the efficacy of a CRISPR-Cas9 approach that uses multiplexed targeting to simultaneously target the viral genome at two or more sites, successfully eliminating the chili leaf curl virus (ChiLCV) genome without the formation of escape mutants [32]. The most effective viral interference occurred when the cotton leaf curl Kokhran virus-Burewala strain (CLCuKoV-Bur) genome was simultaneously targeted at two points [33]. Furthermore, multiplex targeting with CRISPR/Cas9, which simultaneously targets six genes of the cotton leaf curl virus (CLCuV), was more effective in interfering with viral proliferation than individually targeting a single region [41]. These findings suggest that CRISPR/Cas9-based multiplexed targeting may improve the efficiency of viral interference and can be engineered for mixed-virus infections.

Similarly, numerous studies have used CRISPR/Cas9 to produce virus-resistant plants of crop varieties other than *N. benthamiana* and *Arabidopsis*. Recent evidence has demonstrated the simplicity and efficiency of this antiviral system. Two independent experiments successfully used CRISPR/Cas9 to establish a highly extreme resistance to wheat dwarf virus in barley [34] and common wheat [35]. Empirical evidence shows that cleavage by CRISPR-Cas9 leads to a conserved single-nucleotide mutation in the viral genomes of cassava transgenic lines [36]. This assertion was further corroborated by two independent studies that showed that targeting different regions of the tomato yellow leaf curl virus (TYLCV) genome directly using CRISPR/Cas9 or inducible CRISPR/Cas9 reduced viral accumulation in transgenic tomato plants [37,38]. Using an inducible promoter to initiate Cas9 activity presents an opportunity to overcome the potential off-target effects of Cas9 [37]. These findings strongly suggest the efficacy of CRISPR/Cas9 for engineering robust immunity against diverse *Geminiviruses*.

In addition to interference with *Geminiviruses*, robust resistance has been achieved against other DNA virus families using CRISPR/Cas9. Cas9-mediated multiplex targeting of the viral CP sequence results in strong viral resistance against cauliflower mosaic virus (CaMV) [39]. Similarly, Tripathi et al. (2019) demonstrated the potential of CRISPR/Cas9 to interfere with the endogenous banana streak virus (eBSV) by editing viral sequences in bananas [40].

### 3.2. Virus Interference by Editing Plant Host Gene via CRISPR/Cas9

A second strategy for viral resistance involves editing the plant genome using CRISPR/Cas (Figure 1B). Typically, single-stranded positive-sense RNA viruses require hijacking of plant host factors for survival. Hence, the success of viral infection depends on the recruitment of host genes. Modifying host genes involved in the viral life cycle, including replication and movement, could result in the loss of susceptibility, yielding recessive resistance [42]. Numerous studies have documented successful applications for engineering resistance against plant viruses (Table 2).

The eukaryotic translation initiation factor *eIF4E* and its isoforms *eIF(iso)4E* and *eIF4G* are major plant host susceptibility factors [61,62]. As shown in Table 2, the editing of *eIF4E* and its isoforms in plants has been reported in many different publications in recent years. Mutagenesis of *eIF4E* and other isoforms using the CRISPR/Cas9 system, such as mutations in *eIF(iso)4E*, *eIF4G*, *nCBP-1*, and *nCBP-2* in various plants, results in effective and broad-spectrum resistance to RNA viruses, particularly those belonging to *Potyvirus* [43,44,45,46,47,48,49]. Knocking out *eIF(iso)4E* in sugar beet showed that eIF-mediated recessive resistance can be generated against *poleroviruses* in an agronomically important host [50]. Notably, these results further confirmed that the mutations could be transmitted to subsequent generations in cucumber and tomato [43,48]. Thus, the significant role of *eIF4E* in the life cycle of RNA viruses demonstrates the feasibility of using CRISPR/Cas9 to accelerate plant breeding for trait improvement.

Moreover, resistance based on the knockout of the *eIF4E* gene in plants does not consistently confer broad-spectrum protection. This is evidence that *eIF4E*-coding gene knockout has partial resistance to pepper veinal mottle virus (PVMV) and no resistance to other *Potyviruses* [51]. Similarly, knocking out *eIF4E1* using CRISPR/Cas9 base-editing technology led to higher susceptibility to TuMV in *A. thaliana* [52]. Furthermore, when the tomato *SleIF4E1* and *SleIF4E2* genes were knocked out, the mutant plants showed reduced susceptibility to potato virus Y (PVY), but they did not show any effect on other *Potyviruses* [53]. Consequently, Kuroiwa et al. (2022) found that inactivation of *eIF4E2* genes by CRISPR-Cas9 displayed a narrow resistance spectrum to *Potyvirus*, with edited plants showing complete resistance to one isolate of PVMV but only partial resistance to another isolate [54]. Therefore, it is advisable to identify multiple host genes that are targeted by CRISPR/Cas9.

In addition, recent studies have suggested that knocking out other host susceptibility factors also generates resistance to RNA viruses. For instance, targeting the tomato susceptibility gene *SlPelo* resulted in high resistance to TYLCV [55]. Similarly, the disruption of homologous genes of the Chloride channel (*CLC-Nt1*) in *Nicotiana benthamiana* using the CRISPR/Cas9 system contributed to a reduction in PVY replication [56]. Editing at least one allele of the *coilin* gene using a DNA-free approach considerably increased resistance to PVY and tolerance to salt and osmotic stress [63]. Multiplexed targeting of *GmF3H1*, *GmF3H2*, and *GmFNSII-1* via CRISPR/Cas9 in soybean increased isoflavone content and improved resistance to soybean mosaic virus (SMV) [57]. Moreover, recent studies have achieved reliable resistance to barley mild mosaic virus (BaMMV), a member of the *bymovirus* family, by editing the wheat host factor gene *TaPDIL5-1* without any negative effects [58]. Promising results have been reported regarding the identification of the susceptibility gene *Tom1*, which is involved in the multiplication of Tobamoviruses in *Arabidopsis* [64]. The above results offer an efficient target to confer potent resistance to the tomato brown rugose fruit virus (ToBRFV) by utilizing CRISPR/Cas9-mediated genome editing to knockout *TOM1* homologs in tomatoes [59]. Additionally, the knockout of two newly identified susceptibility genes, *NbUbEF1B* and *NbCCR4/NOT3*, results in high-efficiency broad-spectrum resistance to various *Geminiviruses* [60]. These findings underscore the potential of exploiting these susceptibility factors to create novel broad-spectrum resistance in crop plants using the CRISPR/Cas9 system.

### 3.3. Engineering RNA Virus Resistance in Plants via CRISPR/Cas13

Recently, a newly identified CRISPR/Cas13a system was found to cleave single-stranded RNA, conferring resistance to RNA phages [65]. CRISPR/Cas13a contains two highly prokaryotic and eukaryotic nucleotide-binding RNase domains that mediate targeted RNA cleavage [66]. CRISPR/Cas13a specifically knocked down the expression of target genes. The efficiency of gene silencing using CRISPR/Cas13a is comparable to that of RNA interference [18]. One notable advantage of CRISPR/Cas13a-mediated gene silencing is the reduction in off-target effects in both plant and mammalian cells [18].

In addition to CRISPR/FnCas9, the application of CRISPR/Cas13a significantly enhanced the inhibition of plant RNA viruses (Table 3; Figure 1C). Initial research has indicated the potential of the CRISPR/Cas13a system to interfere with TuMV in tobacco. Moreover, a study by Aman et al. (2018) revealed that targeting the HC-Pro region of TuMV was superior for engineering viral resistance compared to targeting the CP region [67]. Subsequently, Zhan et al. (2019) further demonstrated that broad-spectrum resistance to different PVY strains in transgenic potatoes was achieved by sgRNA directing Cas13a to target the conserved sequence of multiple PVY [68]. The results showed a positive correlation between the efficiency of PVY inhibition and the expression levels of Cas13a/sgRNA in transgenic potatoes. Notably, their further study showed that the expression of multiple gRNAs did not significantly influence the efficacy of CRISPR/Cas13a-mediated viral interference in plants [69]. In another study, the CRISPR/Cas13a system also exhibited resistance against RNA viruses, such as the southern rice black-streaked dwarf virus and rice stripe mosaic virus, in monocot plants [70].

Subsequent studies evaluated an array of Cas13 variants to identify an effective version against both single and multiple RNA viruses. Mahas et al. (2019) demonstrated that LwaCas13a, PspCas13b, and CasRx variants exhibited high interference activity against RNA viruses in transient assays, with CasRx displaying robust interference in both transient and stable overexpression assays among the examined variants [71]. Similarly, Cao et al. (2021) verified the efficacy of a CasRx-mediated RNA interference system in attenuating plant RNA virus infection and suppressing target RNA expression [72]. Infiltration of CasRx with a tobacco rattle virus (TRV)-gRNA expression system resulted in lower grapevine virus A accumulation in the model plant *Nicotiana benthamiana* [73]. These findings significantly broaden the potential applications of the CRISPR/Cas13 system in countering viral RNA interference.

Further research has highlighted the potential of Cas13 variants to generate broad-spectrum resistance against mixed RNA viral infections using multiplex strategies to target multiple viral species. Yu et al. (2022) discovered that the variants LwaCas13a and RfxCas13d could facilitate multiplexed targeting of the same virus or simultaneous targeting of multiple RNA viruses in plants. This function is contingent on the Cas13 effector action on pre-gRNAs to produce multiple functional gRNAs [74]. In contrast to this gRNA-processing mechanism, numerous studies have explored alternative multiplex strategies. A recent investigation demonstrated the use of polyvalent guide RNAs (pgRNAs) to target multiple viral sites and further revealed that pgRNAs in complex with RfxCas13d effectively suppressed viral dissemination in *N. benthamiana* [75]. Zhan et al. (2023) showed that an RNA editing system can directly target multiple viral genomes to efficiently suppress dual or triple mixed infections in potatoes by utilizing an endogenous tRNA-processing system to generate multiple functional gRNAs [76]. In summary, these observations suggest that CRISPR/Cas13 has impressive catalytic activity and high specificity, providing a new basis for manipulating multiple viral RNA genomes effectively.

Notably, recent reports have revealed the use of an ortholog of CRISPR/Cas9, FnCas9, derived from the pathogenic bacterium *Francisella novicida*, to target RNA molecules, allowing the modification of the plant RNA virus genome. For instance, Zhang et al. (2018) harnessed the capabilities of CRISPR/FnCas9 to provide resistance against both cucumber mosaic virus (CMV) and tobacco mosaic virus (TMV) in *N. benthamiana* and *Arabidopsis* plants [77]. A comparative analysis of the interference efficiency between LshCas13a and FnCas9 in grapevine established that LshCas13a exhibited superior interference efficiency against grapevine leaf roll-associated virus 3 (GLRaV-3) [78]. Therefore, these studies collectively suggest that the CRISPR/Cas13 system is a promising strategy for imparting durable resistance to RNA viruses in different crop species.

A recent surprising discovery showed that a crRNA designed to guide Cas13 could result in effective gene silencing, even in the absence of the Cas13 protein in three plant species, a phenomenon termed guide-induced gene silencing (GIGS) [79]. This novel GIGS phenomenon offers a flexible approach for RNA reduction, contributing to crop improvement and functional genomics. However, the existence of GIGS remains a subject of debate. In another study, targeting multiple gRNAsystems did not result in GIGS [73].

## 4. Discussion

### 4.1. The Delivery Approachesfor CRISPR/Cas-Based Antivral Resistance

The efficacy of gene targeting depends on the suitable delivery methods of CRISPR/Ca components, and an effective delivery approach is crucial for conferring resistance to viruses in plants. Current approaches to introduce CRISPR/Cas-mediated resistance in plants include *Agrobacterium*-mediated transformation, biolistic bombardment, transient protoplast transfection, and plant virus-based transient delivery vectors. Plasmids, plant viruses, and RNP complexes usually act as carriers for loading functional sequences and proteins into plant cells [80].

*Agrobacterium*-mediated transformation is the most widely used delivery method because of its broad applicability, low economic cost, and suitability for various plants [81]. Transgenic plants generated by stable *Agrobacterium* transformation are of great interest in field trials because of the random insertion of T-DNA into plant genomes. The same concern also occurs in biolistic bombardment and transient protoplast transfection when a large amount of plasmid DNA is used to deliver CRISPR/Cas [82,83,84].

Considerable efforts have been made to develop DNA-free genome editing methods to eliminate the negative public perceptions associated with transgenic plants. *Agrobacterium*-mediated transient expression has been achieved without T-DNA in tomato and potato plants; however, the editing efficiency was lower than that of stable transformation [85]. Some alternative delivery methods, such as biolistic bombardment or transfection of protoplasts coated with ribonucleoprotein (RNP) complexes, have been employed to produce non-transgenic plants. These two delivery methods have greatly improved editing efficiency [86,87]. However, the significant challenges of these approaches are limited to some plant species due to various bottlenecks [88].

Therefore, another CRISPR/Cas delivery method is required for efficient DNA-free genome editing. Plant RNA viruses can act as promising transient delivery vectors for CRISPR/Cas components in a process named virus-induced genome editing (VIGE). Over the last few years, several viral vectors have been engineered to deliver genome-editing components to plant cells, typically like *Geminiviruses* and single-stranded DNA viruses, including BeYDV [89], beet curly top virus (BCTV) [90], and sweet potato leaf curl virus (SPLCV) [91]. All the above examples have shown great improvements in plant genome editing using CRISPR/Cas. In addition, single-stranded RNA viruses, such as tobacco rattle virus (TRV) [92], pea early browning virus (PEBV) [93], and potato virus X (PVX) [94], have also been shown to transport CRISPR-Cas components efficiently and have a higher editing ability.

### 4.2. The Off-Target Effects in CRISPR-Edited Virus Resistance Plants

In plants, CRISPR/Cas9 and CRISPR/Cas13 have been used to interfere with DNA and RNA viruses, respectively. However, using CRISPR/Cas to engineer virus-resistant crops faces significant obstacles. Ji et al. (2018) have shown that constitutive expression of Cas9/sgRNA could cause off-target effects in CRISPR-edited virus resistance plants [95], raising concerns regarding the durability of virus resistance by using CRISPR/Cas9 systems in plants. Nevertheless, several strategies can be adopted to minimize or prevent these off-target effects in CRISPR-edited virus-resistant plants: (i) Adoption of the virus-inducible CRISPR/Cas9 system. These systems have been shown to effectively interfere with viral DNA in transgenic plants. Deep sequencing revealed that transgenic plants do not exhibit off-target effects [95]; (ii) Development of virus-resistant crops using the dead Cas9 (dCas9)-FokI strategy. The wild-type FokI nuclease domain fused with catalytically inactive Cas9 (dCas9) forms the dCas9-FokI complex, which is recruited to adjacent target sites by two different gRNAs [96]. Relative to monomeric Cas9 nucleases, a significant strength of dCas9-FokI is that the off-target effects in human cells are reduced because the dimeric RNA-guided FokI nucleases (RFNs) function as dimers, necessitating two gRNAs for enzymatic activity [96]; (iii) The production of virus-resistant crops using the CRISPR/Cas13 strategy. The RNA intermediates generated in the DNA virus life cycle make CRISPR/Cas13 specificity systems the preferred choice for this application [24,97]. Although off-target effects are also a critical limitation of the CRISPR/Cas13 system, no off-target effects have been detected in the CRISPR/Cas13 editing system to date; and (IV) Off-target effects are the result of mismatches in the gRNA sequence because Cas9 tolerates up to three mismatches between gRNA and genomic DNA [98]. Shorter guide RNAs [99,100] and stricter gRNA design rules [101] are required to solve this off-target problem.

### 4.3. The Evolution of CRISPR-Resistant Plant Viruses

*Geminiviruses* evade CRISPR/Cas9-mediated resistance, posing a challenge to genetic editing. Ali et al. (2016) highlighted the ability of *Geminiviruses* to evade the CRISPR/Cas9 machinery by generating viral variants under the pressure of targeting coding sequences [30]. Furthermore, Mehta et al. (2019) illustrated that transgenic cassava expression of CRISPR/Cas9 could lead to CRISPR-resistant viruses during glasshouse inoculation. Sequencing of viruses in transgenic cassava plants has revealed an abundance of single-nucleotide mutations within the viral genomes [36]. These findings indicate that transgenic plants with CRISPR/Cas9 can exert tremendous selective pressure on plant viruses, ultimately facilitating the evolution of resistant viruses to escape cleavage.

Several potential strategies to prevent or delay the emergence of new viruses are as follows: (i) Selecting conserved regions for targeting by aligning the genomes of diverse strains that are not likely to mutate; (ii) Designing different gRNAs to target multiple sites in the viral genome to reduce the likelihood of generating escape variants. The endogenous tRNA-processing system in plant cells has been tested to simultaneously produce numerous gRNAs, enabling multiplex targeting and improving the editing efficiency of the CRISPR/Cas9 system in plants [102]. The efficiency of large fragment deletions can be further improved by combining multiple gRNAs in the CRISPR/Cas system [103], and (iii) Using other highly efficient RNA-targeting effectors, such as CRISPR/Cas12a and CRISPR/Cas12b, may be beneficial for eliminating viral evolution.

It is also possible that CRISPR/Cas13-mediated viral interference results in the evolution of RNA viruses. However, it remains unclear whether CRISPR/Cas13 can accelerate the evolution of RNA viruses. Fortuitously, multiple targeting combined with CRISPR/Cas13 conferred PVY tolerance to potatoes by simultaneously expressing four gRNAs [69]. Notably, a more potent effect factor has been identified: the ultra-small family of Cas13bt belonging to type VI-B. It exhibited significantly improved knockdown of the target genes compared to the other Cas13 variants. The RNA knockdown efficiency and specificity of the Cas13bt effector were more favorable than those of RNA interference [104].

### 4.4. Comparison of RNA Silencing and CRISPR/Cas Editing Antiviral Strategies

RNA silencing and genome editing are two major antiviral strategies used in plant genetic engineering. RNA silencing has been successfully applied to target various viruses across nearly 30 plant species by inducing sequence-specific inhibition or suppression of viral RNA. This method has shown greater efficacy against RNA viruses than against DNA viruses [14,105]. Resistance based on RNA silencing can be achieved through various approaches, such as sense/antisense RNA, hairpin RNA, and artificial miRNA [106,107]. Recent advances have demonstrated the possibility of simultaneously targeting multiple plant viruses by creating hairpin RNA structures in a single construct, offering a promising strategy for broad-spectrum viral control [108,109]. One of the biggest concerns is the occurrence of viruses that encode viral suppressors of RNA silencing (VSRs) [110]. The rapid development of the CRISPR/Cas editing technique is a new strategy for improving broad-spectrum resistance to plant viruses. Unlike RNA silencing, viruses have not yet evolved the ability to counter CRISPR/Cas-mediated immune responses. Nevertheless, these new systems still have limitations, such as the off-targeting effect and the emergence of escape virus mutants. Furthermore, targeting host susceptibility factors based on the CRISPR/Cas technique may result in resistance to certain isolates [48,49,53,54] and, in one case, unintended yield higher susceptibility to other RNA viruses [52].

### 4.5. CRISPR-Edited Virus-Resistant Plants Status

Engineered CRISPR/Cas-mediated viral resistance in plants can be achieved using various strategies. Direct targeting of the viral genome requires constitutive expression of CRISPR/Cas components; thus, transgenic CRISPR-edited virus-resistant plants fail to gain public acceptance. When host genes are targeted by DNA-free genome editing, CRISPR-Cas transgenes can be transiently expressed or segregated to generate non-transgenic plants. However, virus-related host genes are often involved in essential endogenous processes, such as plant growth and development; thus, targeting host genes may impact host plants, resulting in unexpected drawbacks [111]. Negative perceptions prevent this technology from being used to reap fruits for successful crop breeding. Therefore, it is imperative to identify novel virus-related host genes in addition to the *elF4E* gene, which exhibits minimal effects after mutation. An alternative option involves regulating the content of virus-relevant metabolites like isoflavone through gene editing techniques to achieve resistance against the virus, as commented by Zhang et al. (2020) [57].

### 4.6. The Durability of CRISPR-Mediated Resistance

It is generally considered that recessive resistance acquired by loss-of-function of the host susceptibility factor by targeted mutagenesis is more durable than dominant gene-based resistance because of lower selective pressures for the virus to evolve counter-defense strategies [112]. However, so far, only a limited number of studies have tested the durability of CRISPR-edited virus-resistant plants in subsequent generations [45,61]; thus, the long-term durability of such newly engineered resistance remains to be verified.

## 5. Conclusions and Future Perspective

These recent findings indicate that CRISPR/Cas systems represent a revolutionary advancement in engineering plant resistance to DNA and RNA viruses. The predominant focus has been on CRISPR/Cas9 and CRISPR/Cas13, which offer opportunities for the development of novel antiviral approaches. There is a significant demand for other CRISPR/Cas systems with high specificity and efficacy for interference with plant viruses to address these concerns. Therefore, more efforts should be made to test plant resistance to viruses using CRISPR/Cas technology in the field.

## Figures and Tables

**Table 1:** Summary of CRISPR/Cas9-induced virus resistance by targeting the viral genome in plants.

Plant Species	Comments	Resistance to Viruses	Viruses Genome and Genus	Target Regions of Virus Genome	%Reduction in Viral Titers	Reference
*Arabidopsis thaliana* *N. benthamiana*	SpCas9	Beet severe curly top virus (BSCTV)	ssDNA, *Geminiviruses*	Replicase (Rep)	70–95	[27]
				Coat protein (CP)	20–90	
				Intergenic region (IR)	30–90	
*Nicotiana benthamiana*	SpCas9	Bean yellow dwarf virus (BeYDV)	ssDNA, *Geminiviruses*	Rep binding site (RBS)	NA	[28]
				hairpin, nonanucleotide sequence	71	
				three Rep motifs	78	
	SpCas9	Tomato yellow leaf curl virus (TYLCV)	ssDNA, *Begomovirus*	Intergenic region (IR), CP, RCRII	NA	[29]
	SpCas9	Cotton Leaf Curl Kokhran Virus(CLCuKoV) Merremia mosaic viruses (MeMV) Tomato yellow leaf curl virus (TYLCV)	ssDNA, *Begomovirus*	Coat protein (CP), non-coding IR, Rep RCRII domain	NA	[30]
	SpCas9	Cotton Leaf Curl Multan virus (CLCuMuV)	ssDNA, *Geminiviruses*	IR region, the C1 coding region	NA	[31]
	SpCas9	Chilli leaf curl virus (ChiLCV)	ssDNA, *Begomoviruses*	IR, overlapping regions between V2 and V1 genes, overlapping regions between C1 and C4 genes	60–90	[32]
	SpCas9	Cotton leaf curl Kokhran virus -Burewala strain (CLCuKoV)	ssDNA, *Geminiviruses*	Intergenic region (IR)	NA	[33]
*Hordeum vulgare* (barley)	SpCas9	Wheat dwarf virus (WDV)	ssDNA, *Geminiviridae*	MP, CP, Rep/RepA, LIR region	NA	[34]
*Triticum aestivum L*. (wheat)	SpCas9	Wheat dwarf virus (WDV)	ssDNA, *Geminiviridae*	CP	25–55	[35]
				MP	25–55	
				LIR	48–97	
*Manihot esculenta* (Cassava)	SpCas9	African cassava mosaic virus (ACMV)	ssDNA, *Begomovirus*	Viral AC2 gene, AC3 gene	0	[36]
*Solanum lycopersicum* (tomato)	SpCas9	Tomato yellow leaf curl virus (TYLCV)	ssDNA, *Begomovirus*	CP	69	[37]
				Rep	74	
*Solanum lycopersicum* (tomato)	SpCas9	Tomato yellow leaf curl virus (TYLCV)	ssDNA, *Begomovirus*	IR	60	[38]
				CP	80	
*Arabidopsis thaliana*	SpCas9	Cauliflower mosaic virus (CaMV)	dsDNA, *Brassicaceae*	CP	20–52	[39]
*Musa balbisiana* (banana)	SpCas9	Banana streak virus (eBSV)	dsDNA, *Caulimoviridae*	Three open-reading frames	NA	[40]

**Table 2:** Studies based on CRISPR/Cas9 strategies for targeting plant host genes leading to plant resistance against virus infections.

Host Plant	Resistance to Viruses	Viruses Genus	Targeted Host Genes	% Mutations of the Host Gene	Reference
*Arabidopsis thaliana*	Turnip mosaic virus (TuMV)	*Potyviruses*	*eIF(iso)4E*	NA	[43]
*Cucumis sativus* (Cucumber)	Cucumber vein yellowing virus (CVYV), Zucchini yellow mosaic virus (ZYMV), Papaya ring spot mosaic virus-W(PRSV-W)	*Ipomovirus* *Potyviruses*	*eIF4E*	NA	[44]
*Oryza sativa* (rice)	Rice tungro spherical virus (RTSV)	*Tospovirus*	*eIF4G*	59	[45]
*Manihot esculenta* (Cassava)	Cassava brown streak virus (CBSV)	*Ipomovirus*	*nCBP-1* *nCBP-2*	78	[46]
*Arabidopsis thaliana*	Clover yellow vein virus (ClYVV)	*Potyviruses*	*eIF4E1*	31	[47]
*Solanum lycopersicum* (tomato)	Pepper mottle virus (PepMoV)	*Potyviruses*	*eIF4E1*	NA	[48]
*Solanum lycopersicum* (tomato)	Cucumber mosaic virus (CMV) Potato virus Y (PVY)	*Cucumovirus* *Potyvirus*	*eIF4E1*	NA	[49]
*Beta vulgaris* (sugar beet)	Beet chlorosis virus	*poleroviruses*	*eIF(iso)4E*	NA	[50]
*Solanum lycopersicum* (tomato)	pepper veinal mottle virus (PVMV)	*Potyviruses*	*eIF4E2*	NA	[51]
*Arabidopsis thaliana*	Clover yellow vein virus (ClYVV)	*Potyvirus*	*eIF4E1*	50	[52]
*Solanum lycopersicum* (tomato)	Potato virus Y (PVY)	*Potyvirus*	*SleIF4E1* *SleIF4E2*	NA	[53]
*Solanum lycopersicum* (cherry tomato)	Pepper veinal mottle virus (PVMV)	*Potyvirus*	*eIF4E2*	NA	[54]
*Solanum lycopersicum* (tomato)	Tomato yellow leaf curl virus	*Begomovirus*	*SlPelo*	10	[55]
*Nicotiana benthamiana*	Potato virus Y (PVY)	*Potyviruses*	*CLC-Nt1*	NA	[56]
*Glycine max* (soya bean)	Soybean mosaic virus (SMV)	*Potyvirus*	*GmF3H1*	55.56	[57]
			*GmF3H2*	48.15	
			*GmFNSII-1*	81.48	
*Triticum aestivum* (hexaploid wheat)	Barley yellow mosaic virus (BaYMV)	*Bymoviruses*	*TaPDIL5-1*	13.1–14.3	[58]
*Solanum lycopersicum* (tomato)	Tomato brown rugose fruit virus (ToBRFV)	*Tobamovirus*	*TOM1*	NA	[59]
*Nicotiana benthamiana*	Tomato yellow leaf curl China virus (TYLCCNV)	*Geminiviruses*	*NbUbEF1B NbCCR4/NOT3*	NA	[60]

**Table 3:** Summary of applications of CRISPR/Cas13 technology for RNA virus resistance.

Plant Species	Comments	Resistance to Viruses	Viruses Genome and Genus	Target Regions	Reference
*Nicotiana benthamiana*	LshCas13a	Turnip Mosaic Virus (TuMV)	ssRNA, *Potyviruses*	HC-Pro, GFP, coat protein (CP)	[67]
*Solanum tuberosum* (potato)	LshCas13a	Potato virus Y (PVY)	ssRNA, *Potyviruses*	P3, CI, NIb, CP regions	[68]
*Solanum tuberosum* (potato)	LshCas13a	Potato virus Y (PVY)	ssRNA, *Potyviruses*	P3, CI, NIb, CP regions	[69]
*Oryza sativa* (rice)	LshCas13a	Southern rice black-streaked dwarf virus (SRBSDV) Rice Stripe Mosaic Virus (RSMV)	dsRNA, *Fijivirus* ssRNA, *Cytorhabdovirus*		[70]
*Nicotiana benthamiana*	LwaCas13a PspCas13b CasRx	Turnip Mosaic Virus (TuMV) tobacco mosaic virus (TMV) Potato virus X (PVX)	ssRNA, *Potyviruses* ssRNA, *Tobamovirus* ssRNA, *Potexvirus*	GFP, CP, Rep, HC-Pro	[71]
*Nicotiana benthamiana*	CasRx	Turnip mosaic virus (TuMV) Tobacco mosaic virus (TMV) Cucumber mosaic virus (CMV)	ssRNA, *Potyviruses* ssRNA, *Tobamovirus* ssRNA, *Cucumovirus*	GFP, HC-Pro	[72]
*Nicotiana benthamiana*	CasRx	grapevine virus A	ssRNA, *Vitivirus*	CP	[73]
*Ipomoea batatas* (sweet potato)	LwaCas13a RfxCas13d PspCas13b LshCas13a	Sweet potato chlorotic stunt virus (SPCSV)	ssRNA, *Crinivirus*	SPCSV-RNase3	[74]
*Nicotiana benthamiana*	RfxCas13d	Tobacco mosaic virus (TMV)	ssRNA, *Tobamovirus*	viral replicase gene, movement protein (MP) gene	[75]
*Solanum tuberosum* (potato)	RfxCas13d	Potato leaf roll virus(PLRV) Potato virus Y (PVY) Potato virus X (PVX) Potato virus S (PVS)	ssRNA, *Polerovirus* ssRNA, *Potyviruses* ssRNA, *Potexvirus* ssRNA, *Carlavirus*	CP	[76]
*Nicotiana benthamiana* *Arabidopsis*	FnCas9	Cucumber mosaic virus (CMV) Tobacco mosaic virus (TMV)	ssRNA, *Cucumovirus* ssRNA, *Tobamovirus*	1A, CP, 3′UTR-A	[77]
*Vitis vinifera* (grapevine)	LshCas13a, FnCas9	Grapevine leafroll-associated virus-3 (GLRaV-3)	ssRNA, *Closteroviridae*	5 kDa protein (p5), heat stimulated protein 70 (Hsp70), heat stimulated protein 90 (Hsp90h), CP, minor coat protein (CPm)	[78]

## References

[1] Jinek M., Chylinski K., Fonfara I., Hauer M., Doudna J.A., Charpentier E. (2012). A programmable dual-RNA-guided DNA endonuclease in adaptive bacterial immunity. Science.

[2] Makarova K.S., Wolf Y.I., Alkhnbashi O.S., Costa F., Shah S.A., Saunders S.J., Barrangou R., Brouns S.J., Charpentier E., Haft D.H. (2015). An updated evolutionary classification of CRISPR-Cas systems. Nat. Rev. Microbiol..

[3] Shmakov S., Smargon A., Scott D., Cox D., Pyzocha N., Yan W., Abudayyeh O.O., Gootenberg J.S., Makarova K.S., Wolf Y.I. (2017). Diversity and evolution of class 2 CRISPR-Cas systems. Nat. Rev. Microbiol..

[4] Chen K., Wang Y., Zhang R., Zhang H., Gao C. (2019). CRISPR/Cas genome editing and precision plant breeding in agriculture. Annu. Rev. Plant Biol..

[5] Zhu H., Li C., Gao C. (2020). Applications of CRISPR-Cas in agriculture and plant biotechnology. Nat. Rev. Mol. Cell Biol..

[6] Mahy B.W.J., Regenmortel M.H.V.V. (2009). Desk Encyclopedia of Plant and Fungal Virology.

[7] Boualem A., Dogimont C., Bendahmane A. (2016). The battle for survival between viruses and their host plants. Curr. Opin. Virol..

[8] Zaidi S.S., Tashkandi M., Mansoor S., Mahfouz M.M. (2016). Engineering plant immunity: Using CRISPR/Cas9 to generate virus resistance. Front. Plant. Sci..

[9] Koonin E.V., Krupovic M., Agol V.I. (2021). The Baltimore Classification of Viruses 50 Years Later: How Does It Stand in the Light of Virus Evolution?. Microbiol. Mol. Biol. Rev..

[10] Gilbertson R.L., Batuman O., Webster C.G., Adkins S. (2015). Role of the insect supervectors *bemisia tabaci* and *frankliniella occi-dentalis* in the emergence and global spread of plant viruses. Annu. Rev. Virol..

[11] Revers F., Garcia J.A. (2015). Molecular Biology of *Potyviruses*. Adv. Virus. Res..

[12] Chaudhary K. (2018). CRISPR/Cas13a targeting of RNA virus in plants. Plant Cell Rep..

[13] Sett S., Prasad A., Prasad M. (2022). Resistance genes on the verge of plant-virus interaction. Trends Plant Sci..

[14] Zhao Y., Yang X., Zhou G., Zhang T. (2020). Engineering plant virus resistance: From RNA silencing to genome editing strategies. Plant Biotechnol. J..

[15] Shin R., Han J.H., Lee G.J., Peak K.H. (2002). The potential use of a viral coat protein gene as a transgene screening marker and multiple virus resistance of pepper plants coexpressing coat proteins of cucumber mosaic virus and tomato mosaic virus. Transgenic Res..

[16] Manghwar H., Lindsey K., Zhang X., Jin S. (2019). CRISPR/Cas System: Recent Advances and Future Prospects for Genome Editing. Trends Plant Sci..

[17] Yan W.X., Hunnewell P., Alfonse L.E., Carte J.M., Keston-Smith E., Sothiselvam S., Garrity A.J., Chong S., Makarova K.S., Koonin E.V. (2019). Functionally diverse type V CRISPR-Cas systems. Science.

[18] Abudayyeh O.O., Gootenberg J.S., Essletzbichler P., Han S., Joung J., Belanto J.J., Verdine V., Cox D.B.T., Kellner M.J., Regev A. (2017). RNA targeting with CRISPR-Cas13. Nature.

[19] Cox D.B.T., Gootenberg J.S., Abudayyeh O.O., Franklin B., Kellner M.J., Joung J., Zhang F. (2017). RNA editing with CRISPR-Cas13. Science.

[20] Yang H., Patel D.J. (2024). Structures, mechanisms and applications of RNA-centric CRISPR-Cas13. Nat. Chem. Biol..

[21] Marraffini L.A. (2015). CRISPR-Cas immunity in prokaryotes. Nature.

[22] Zhang D., Li Z., Li J. (2015). Genome editing: New antiviral weapon for plants. Nat. Plants.

[23] McCarty N.S., Graham A.E., Studená L., Ledesma-Amaro R. (2020). Multiplexed CRISPR technologies for gene editing and transcriptional regulation. Nat. Commun..

[24] Mahas A., Mahfouz M. (2018). Engineering virus resistance via CRISPR-Cas systems. Current. Opin. Virol..

[25] Ji X., Wang D., Gao C. (2019). CRISPR editing-mediated antiviral immunity: A versatile source of resistance to combat plant virus infections. Sci. China Life Sci..

[26] Hadidi A., Flores R., Candresse T., Barba M. (2016). Next-Generation Sequencing and Genome Editing in Plant Virology. Front. Microbiol..

[27] Ji X., Zhang H., Zhang Y., Wang Y., Gao C. (2015). Establishing a CRISPR-Cas-like immune system conferring DNA virus resistance in plants. Nat. Plants.

[28] Baltes N.J., Hummel A.W., Konecna E., Cegan R., Bruns A.N., Bisaro D.M., Voytas D.F. (2015). Conferring resistance to geminiviruses with the CRISPR-Cas prokaryotic immune system. Nat. Plants.

[29] Ali Z., Abulfaraj A., Idris A., Ali S., Tashkandi M., Mahfouz M.M. (2015). CRISPR/Cas9-mediated viral interference in plants. Genome Biol..

[30] Ali Z., Ali S., Tashkandi M., Zaidi S.S., Mahfouz M.M. (2016). CRISPR/Cas9-mediated immunity to Geminiviruses: Differential interference and evasion. Sci. Rep..

[31] Yin K., Han T., Xie K., Zhao J., Song J., Liu Y. (2019). Engineer complete resistance to Cotton Leaf Curl Multan virus by the CRISPR/Cas9 system in *Nicotiana benthamiana*. Phytopathol. Res..

[32] Roy A., Zhai Y., Ortiz J., Neff M., Mandal B., Mukherjee S., Pappu H. (2019). Multiplexed editing of a begomovirus genome restricts escape mutant formation and disease development. PLoS ONE.

[33] Hamza M., Khan M.Z., Mustafa R., Kamal H., Hussain A., Mansoor S., Amin I. (2021). Engineering Resistance Against Cotton Leaf Curl Kokhran Virus-Burewala Strain Using CRISPR-Cas9 system in Nicotiana Benthamiana. Res. Sq..

[34] Kis A., Hamar É., Tholt G., Bán R., Havelda Z. (2019). Creating highly efficient resistance against wheat dwarf virus in barley by employing CRISPR/Cas9 system. Plant Biotechnol. J..

[35] Yuan X., Xu K., Yan F., Liu Z., Spetz C., Zhou H., Wang X., Jin H., Wang X., Liu Y. (2024). CRISPR/Cas9-Mediated Resistance to Wheat Dwarf Virus in Hexaploid Wheat (*Triticum aestivum* L.). Viruses.

[36] Mehta D., Sturchler A., Anjanappa R.B., Zaidi S.S., Hirsch-Hoffmann M., Gruissem W., Vanderschuren H. (2019). Linking CRISPR-Cas9 interference in cassava to the evolution of editing-resistant *geminiviruses*. Genome Biol..

[37] Ghorbani Faal P., Farsi M., Seifi A., Mirshamsi Kakhki A. (2020). Virus-induced CRISPR-Cas9 system improved resistance against tomato yellow leaf curl virus. Mol. Biol. Rep..

[38] Tashkandi M., Ali Z., Aljedaani F., Shami A., Mahfouz M.M. (2018). Engineering resistance against tomato yellow leaf curl virus via the CRISPR/Cas9 system in tomato. Plant Signal Behav..

[39] Liu H., Soyars C.L., Li J., Fei Q., He G., Peterson B.A., Meyers B.C., Nimchuk Z.L., Wang X. (2018). CRISPR/Cas9-mediated resistance to cauliflower mosaic virus. Plant Direct..

[40] Tripathi J.N., Ntui V.O., Ron M., Muiruri S.K., Britt A., Tripathi L. (2019). CRISPR/Cas9 editing of endogenous banana streak virus in the B genome of *Musa* spp. overcomes a major challenge in banana breeding. Commun. Biol..

[41] Mubarik M.S., Wang X., Khan S.H., Ahmad A., Khan Z., Amjid M.W., Razzaq M.K., Ali Z., Azhar M.T. (2021). Engineering broad-spectrum resistance to cotton leaf curl disease by CRISPR-Cas9 based multiplex editing in plants. GM Crops Food.

[42] Gómez P., Rodríguez-Hernández A.M., Moury B., Aranda M.A. (2009). Genetic resistance for the sustainable control of plant virus diseases: Breeding, mechanisms and durability. Eur. J. Plant Pathol..

[43] Pyott D.E., Sheehan E., Molnar A. (2016). Engineering of CRISPR/Cas9-mediated potyvirus resistance in transgene-free Arabidopsis plants. Mol. Plant Pathol..

[44] Chandrasekaran J., Brumin M., Wolf D., Leibman D., Klap C., Pearlsman M., Sherman A., Arazi T., Gal-On A. (2016). Development of broad virus resistance in non-transgenic cucumber using CRISPR/Cas9 technology. Mol. Plant Pathol..

[45] Macovei A., Sevilla N.R., Cantos C., Jonson G.B., Slamet-Loedin I., Cermak T., Voytas D.F., Choi I.R., Chadha-Mohanty P. (2018). Novel alleles of rice *eIF4G* generated by CRISPR/Cas9-targeted mutagenesis confer resistance to *Rice tungro spherical virus*. Plant Biotechnol. J..

[46] Gomez M.A., Lin Z.D., Moll T., Chauhan R.D., Hayden L., Renninger K., Beyene G., Taylor N.J., Carrington J.C., Staskawicz B.J. (2019). Simultaneous CRISPR/Cas9 mediated editing of cassava *eIF4E* isoforms *nCBP-1* and *nCBP-2* reduces cassava brown streak disease symptom severity and incidence. Plant Biotechnol. J..

[47] Bastet A., Zafirov D., Giovinazzo N., Guyon-Debast A., Nogue F., Robaglia C., Gallois J.L. (2019). Mimicking natural polymorphism in eIF4E by CRISPR-Cas9 base editing is with associated resistance to potyviruses. Plant Biotechnol. J..

[48] Yoon Y.J., Venkatesh J., Lee J.H., Kim J., Lee H.E., Kim D.S., Kang B.C. (2020). Genome editing of eIF4E1 in tomato confers resistance to Pepper Mottle virus. Front. Plant Sci..

[49] Atarashi H., Jayasinghe W.H., Kwon J., Kim H., Taninaka Y., Igarashi M., Ito K., Yamada T., Masuta C., Nakahara K.S. (2020). Artificially edited alleles of the eukaryotic translation initiation factor 4E1 gene differentially reduce susceptibility to cucumber mosaic virus and potato virus Y in tomato. Front. Microbiol..

[50] Rollwage L., Van Houtte H., Hossain R., Wynant N., Willems G., Varrelmann M. (2024). Recessive resistance against beet chlorosis virus is conferred by the eukaryotic translation initiation factor (iso)4E in *Beta vulgaris*. Plant Biotechnol. J..

[51] Moury B., Lebaron C., Szadkowski M., Ben Khalifa M., Girardot G., Bolou Bi B.A., Koné D., Nitiema L.W., Fakhfakh H., Gallois J.L. (2020). Knock-out mutation of eukaryotic initiation factor 4E2 (eIF4E2) confers resistance to pepper veinal mottle virus in tomato. Virology.

[52] Zafirov D., Giovinazzo N., Bastet A., Gallois J.L. (2021). When a knockout is an Achilles’ heel: Resistance to one potyvirus species triggers hypersusceptibility to another one in Arabidopsis thaliana. Mol. Plant Pathol..

[53] Kumar S., Abebie B., Kumari R., Kravchik M., Shnaider Y., Leibman D., Bornstein M., Gaba V., Gal-On A. (2022). Development of PVY resistance in tomato by knockout of host eukaryotic initiation factors by CRISPR-Cas9. Phytoparasitica.

[54] Kuroiwa K., Thenault C., Nogué F., Perrot L., Mazier M., Gallois J.L. (2022). CRISPR-based knock-out of eIF4E2 in a cherry tomato background successfully recapitulates resistance to pepper veinal mottle virus. Plant Sci..

[55] Pramanik D., Shelake R.M., Park J., Kim M.J., Hwang I., Park Y., Kim J.Y. (2021). CRISPR/Cas9-Mediated Generation of Pathogen-Resistant Tomato against Tomato Yellow Leaf Curl Virus and Powdery Mildew. Int. J. Mol. Sci..

[56] Sun H., Shen L., Qin Y., Liu X., Hao K., Li Y., Wang J., Yang J., Wang F. (2018). *CLC-Nt1* affects potato virus Y infection via regulation of endoplasmic reticulum luminal Ph. New Phytol..

[57] Zhang P., Du H., Wang J., Pu Y., Yang C., Yan R., Yang H., Cheng H., Yu D. (2020). Multiplex CRISPR/Cas9-mediated metabolic engineering increases soya bean isoflavone content and resistance to soya bean mosaic virus. Plant Biotechnol. J..

[58] Kan J., Cai Y., Cheng C., Jiang C., Jin Y., Yang P. (2022). Simultaneous editing of host factor gene *TaPDIL5-1* homoeoalleles confers wheat yellow mosaic virus resistance in hexaploid wheat. New Phytol..

[59] Ishikawa M., Yoshida T., Matsuyama M., Kouzai Y., Kano A., Ishibashi K. (2022). Tomato brown rugose fruit virus resistance generated by quadruple knockout of homologs of *Tobamovirus multiplication1* in tomato. Plant Physiol..

[60] Li H., Gong P., Xu X., Zhou X., Li F. (2024). Knockout of the virus replication-related genes *UbEF1B* and *CCR4/NOT3* by CRISPR/Cas9 confers high-efficiency and broad-spectrum resistance to *geminiviruses* in *Nicotiana benthamiana*. Plant Biotechnol. J..

[61] Sanfacon H. (2015). Plant translation factors and virus resistance. Viruses.

[62] Borrelli V.M.G., Brambilla V., Rogowsky P., Marocco A., Lanubile A. (2018). The enhancement of plant disease resistance using CRISPR/Cas9 technology. Front. Plant Sci..

[63] Makhotenko A.V., Khromov A.V., Snigir E.A., Makarova S.S., Makarov V.V., Suprunova T.P., Kalinina N.O., Taliansky M.E. (2019). Functional analysis of *Coilin* in virus resistance and stress tolerance of potato *solanum tuberosum* using CRISPR-Cas9 Editing. Dokl. Biochem. Biophys..

[64] Ishikawa M., Obata F., Kumagai T., Ohno T. (1991). Isolation of mutants of Arabidopsis thaliana in which accumulation of tobacco mosaic virus coat protein is reduced to low levels. Mol. Gen. Genet..

[65] Abudayyeh O.O., Gootenberg J.S., Konermann S., Joung J., Slaymaker I.M., Cox D.B., Shmakov S., Makarova K.S., Semenova E., Minakhin L. (2016). C2c2 is a single-component programmable RNA-guided RNA-targeting CRISPR effector. Science.

[66] East-Seletsky A., O’Connell M.R., Knight S.C., Burstein D., Cate J.H., Tjian R., Doudna J.A. (2016). Two distinct RNase activities of CRISPR-C2c2 enable guide-RNA processing and RNA detection. Nature.

[67] Aman R., Ali Z., Butt H., Mahas A., Aljedaani F., Khan M.Z., Ding S., Mahfouz M. (2018). RNA virus interference via CRISPR/Cas13a system in plants. Genome Biol..

[68] Zhan X., Zhang F., Zhong Z., Chen R., Wang Y., Chang L., Bock R., Nie B., Zhang J. (2019). Generation of virus-resistant potato plants by RNA genome targeting. Plant Biotechnol. J..

[69] Zhan X., Tu Z., Song W., Nie B., Li S., Zhang J., Zhang F. (2023). Cas13a-based multiplex RNA targeting for potato virus Y. Planta.

[70] Zhang T., Zhao Y., Ye J., Cao X., Xu C., Chen B., An H., Jiao Y., Zhang F., Yang X. (2019). Establishing CRISPR/Cas13a immune system conferring RNA virus resistance in both dicot and monocot plants. Plant Biotechnol. J..

[71] Mahas A., Aman R., Mahfouz M. (2019). CRISPR-Cas13d mediates robust RNA virus interference in plants. Genome Biol..

[72] Cao Y., Zhou H., Zhou X., Li F. (2021). Conferring resistance to plant RNA viruses with the CRISPR/CasRx system. Virol. Sin..

[73] Spencer K.P., Burger J.T., Campa M. (2023). CRISPR-based resistance to grapevine virus A. Front. Plant. Sci..

[74] Yu Y., Pan Z., Wang X., Bian X., Wang W., Liang Q., Kou M., Ji H., Li Y., Ma D. (2022). Targeting of SPCSV-RNase3 via CRISPR-Cas13 confers resistance against sweet potato virus disease. Mol. Plant. Pathol..

[75] Bagchi R., Tinker-Kulberg R., Salehin M., Supakar T., Chamberlain S., Ligaba-Osena A., Josephs E.A. (2022). Polyvalent guide RNAs for CRISPR antivirals. iScience.

[76] Zhan X., Liu W., Nie B., Zhang F., Zhang J. (2023). Cas13d-mediated multiplex RNA targeting confers a broad-spectrum resistance against RNA viruses in potato. Commun. Biol..

[77] Zhang T., Zheng Q., Yi X., An H., Zhao Y., Ma S., Zhou G. (2018). Establishing RNA virus resistance in plants by harnessing CRISPR immune system. Plant Biotechnol. J..

[78] Jiao B., Hao X., Liu Z., Liu M., Wang J., Liu L., Liu N., Song R., Zhang J., Fang Y. (2022). Engineering CRISPR immune systems conferring GLRaV-3 resistance in grapevine. Hortic. Res..

[79] Sharma V.K., Marla S., Zheng W., Mishra D., Huang J., Zhang W., Morris G.P., Cook D.E. (2022). CRISPR guides induce gene silencing in plants in the absence of Cas. Genome Biol..

[80] Amirkhanov R.N., Stepanov G.A. (2019). Systems of Delivery of CRISPR/Cas9 Ribonucleoprotein Complexes for Genome Editing. Russ. J. Bioorg. Chem..

[81] Sandhya D., Jogam P., Allini V.R., Abbagani S., Alok A. (2020). The present and potential future methods for delivering CRISPR/Cas9 components in plants. J. Genet. Eng. Biotechnol..

[82] Kim H., Kim S.T., Ryu J., Kang B.C., Kim J.S., Kim S.G. (2017). CRISPR/Cpf1-mediated DNA-free plant genome editing. Nat. Commun..

[83] Liang Z., Chen K., Li T., Zhang Y., Wang Y., Zhao Q., Liu J., Zhang H., Liu C., Ran Y. (2017). Efficient DNA-free genome editing of bread wheat using CRISPR/Cas9 ribonucleoprotein complexes. Nat. Commun..

[84] Andersson M., Turesson H., Nicolia A., Fält A.S., Samuelsson M., Hofvander P. (2017). Efficient targeted multiallelic mutagenesis in tetraploid potato (*Solanum tuberosum*) by transient CRISPR-Cas9 expression in protoplasts. Plant Cell Rep..

[85] Veillet F., Perrot L., Chauvin L., Kermarrec M.P., Guyon-Debast A., Chauvin J.E., Nogué F., Mazier M. (2019). Transgene-free genome editing in tomato and potato plants using *Agrobacterium*-mediated delivery of a CRISPR/Cas9 cytidine base editor. Int. J. Mol. Sci..

[86] Lin C.S., Hsu C.T., Yang L.H., Lee L.Y., Fu J.Y., Cheng Q.W., Wu F.H., Hsiao H.C., Zhang Y., Zhang R. (2018). Application of protoplast technology to CRISPR/Cas9 mutagenesis: From single-cell mutation detection to mutant plant regeneration. Plant Biotechnol. J..

[87] Kuluev B.R., Gumerova G.R., Mikhaylova E.V., Gerashchenkov G.A., Rozhnova N.A., Vershinina Z.R., Khyazev A.V., Matniyazov R.T., Baymiev A.K., Baymiev A.K. (2019). Delivery of CRISPR/Cas Components into higher plant cells for genome editing. Russ. J. Plant Physiol..

[88] Uranga M., Daròs J.A. (2023). Tools and targets: The dual role of plant viruses in CRISPR-Cas genome editing. Plant Genome.

[89] Butler N.M., Baltes N.J., Voytas D.F., Douches D.S. (2016). *Geminivirus*-mediated genome editing in potato (*Solanum tuberosum* L.) using sequence-specific nucleases. Front. Plant Sci..

[90] Eini O., Schumann N., Niessen M., Varrelmann M. (2022). Targeted mutagenesis in plants using Beet curly top virus for efficient delivery of CRISPR/Cas12a components. New Biotechnol..

[91] Yu Y., Wang X., Sun H., Liang Q., Wang W., Zhang C., Bian X., Cao Q., Li Q., Xie Y. (2020). Improving CRISPR-Cas-mediated RNA targeting and gene editing using SPLCV replicon-based expression vectors in *Nicotiana benthamiana*. Plant Biotechnol. J..

[92] Ghoshal B., Vong B., Picard C.L., Feng S., Tam J.M., Jacobsen S.E. (2020). A viral guide RNA delivery system for CRISPR-based transcriptional activation and heritable targeted DNA demethylation in Arabidopsis thaliana. PLoS Genet..

[93] Ali Z., Eid A., Ali S., Mahfouz M.M. (2018). Pea early-browning virus-mediated genome editing via the CRISPR/Cas9 system in *Nicotiana benthamiana* and *Arabidopsis*. Virus Res..

[94] Uranga M., Aragonés V., García A., Mirabel S., Gianoglio S., Presa S., Granell A., Pasin F., Daròs J.A. (2024). RNA virus-mediated gene editing for tomato trait breeding. Hortic. Res..

[95] Ji X., Si X., Zhang Y., Zhang H., Zhang F., Gao C. (2018). Conferring DNA virus resistance with high specificity in plants using virus-inducible genome-editing system. Genome Biol..

[96] Tsai S.Q., Wyvekens N., Khayter C., Foden J.A., Thapar V., Reyon D., Goodwin M.J., Aryee M.J., Joung J.K. (2014). Dimeric CRISPR RNA-guided FokI nucleases for highly specific genome editing. Nat. Biotechnol..

[97] Roossinck M.J. (2003). Plant RNA virus evolution. Curr. Opin. Microbiol..

[98] Wang H., La Russa M., Qi L.S. (2016). CRISPR/Cas9 in genome editing and beyond. Annu. Rev. Biochem..

[99] Fu Y., Sander J.D., Reyon D., Cascio V.M., Joung J.K. (2014). Improving CRISPR-Cas nuclease specificity using truncated guide RNAs. Nat. Biotechnol..

[100] Coelho M.A., De Braekeleer E., Firth M., Bista M., Lukasiak S., Cuomo M.E., Taylor B.J.M. (2020). CRISPR GUARD protects off-target sites from Cas9 nuclease activity using short guide RNAs. Nat. Commun..

[101] Doench J.G., Fusi N., Sullender M., Hegde M., Vaimberg E.W., Donovan K.F., Smith I., Tothova Z., Wilen C., Orchard R. (2016). Optimized sgRNA design to maximize activity and minimize off-target effects of CRISPR-Cas9. Nat. Biotechnol..

[102] Xie K., Minkenberg B., Yang Y. (2015). Boosting CRISPR/Cas9 multiplex editing capability with the endogenous tRNA-processing system. Proc. Natl. Acad. Sci. USA.

[103] Han J., Zhang J., Chen L., Shen B., Zhou J., Hu B., Du Y., Tate P.H., Huang X., Zhang W. (2014). Efficient in vivo deletion of a large imprinted lncRNA by CRISPR/Cas9. RNA Biol..

[104] Kannan S., Altae-Tran H., Jin X., Madigan V.J., Oshiro R., Makarova K.S., Koonin E.V., Zhang F. (2022). Compact RNA editors with small Cas13 proteins. Nat. Biotechnol..

[105] Ali A., Shahbaz M., Ölmez F., Fatima N., Umar U.U.D., Ali M.A., Akram M., Seelan J.S.S., Baloch F.S. (2024). RNA interference: A promising biotechnological approach to combat plant pathogens, mechanism and future prospects. World J. Microbiol. Biotechnol..

[106] Simón-Mateo C., García J.A. (2011). Antiviral strategies in plants based on RNA silencing. Biochim. Biophys. Acta.

[107] Taliansky M., Samarskaya V., Zavriev S.K., Fesenko I., Kalinina N.O., Love A.J. (2021). RNA-based technologies for engineering plant virus resistance. Plants.

[108] Azizi A., Verchot J., Moieni A., Shams-Bakhsh M. (2020). Efficient silencing gene construct for resistance to multiple common bean (*Phaseolus vulgaris* L.) viruses. 3 Biotech.

[109] Li C., Wu J., Fu S., Xu Y., Wang Y., Yang X., Lan Y., Lin F., Du L., Zhou T. (2024). Development of a transgenic rice line with strong and broad resistance against four devastating rice viruses through expressing a single hairpin RNA construct. Plant Biotechnol. J..

[110] Duan C.G., Wang C.H., Guo H.S. (2012). Application of RNA silencing to plant disease resistance. Silence.

[111] Hoffie R.E., Otto I., Perovic D., Budhagatapalli N., Habekuß A., Ordon F., Kumlehn J. (2021). Targeted knockout of eukaryotic translation initiation factor 4E confers bymovirus resistance in winter barley. Front. Genome Ed..

[112] de Ronde D., Butterbach P., Kormelink R. (2014). Dominant resistance against plant viruses. Front. Plant Sci..

